# Trust and website conversion in consumer responses to green product purchasing: A new perspective through the lens of innovative website Design's technology integration

**DOI:** 10.1016/j.heliyon.2023.e23764

**Published:** 2023-12-17

**Authors:** Wutthiya Aekthanate Srisathan, Chavis Ketkaew, Narissara Jantuma, Phaninee Naruetharadhol

**Affiliations:** a International College, Khon Kaen University, Khon Kaen, 40002, Thailand; b Center for Sustainable Innovation and Society, Khon Kaen, 40002, Thailand; c Hincks Centre for Entrepreneurship Excellence, Munster Technological University, Cork, Ireland

**Keywords:** Technology integration of innovative website design, Conversion of consumer response, User trust, Anticipated website usability, Anticipated website quality requirement

## Abstract

This study aims to investigate the role of trust and website conversion in consumer responses to green product purchasing, with a new perspective through the lens of innovative website design's technology integration. To achieve this objective, an online survey was conducted with a sample of 384 consumers in Thailand. The results reveal that user trust in innovative website design are significant predictor website conversion in consumer responses to green product purchasing. Innovative website design's technology integration positively influences anticipated website usability and anticipated website quality requirements, which, in turn, enhances trust and leads to increased consumer conversion to green product purchasing. Moreover, anticipated website quality requirement acts as a partial mediator in the relationship between technology integration of innovative website design and user trust in innovative website design. The relationship between user trust in innovative website design and conversion of consumer response to green product purchase is notably strong (β = 0.203, p-value = 0.014), with user trust accounting for roughly 57.1 % of the variability in consumer conversion ($R^{2}=0.571$). This substantial link suggests that in the broader context, enhancing elements that build trust within a website's design is crucial as it influences over half of the consumer's decision-making process related to green product purchases. Essentially, for industries and markets centered around eco-friendly products, investing in strategies to bolster user trust in their digital platforms could significantly drive consumer response and engagement. These findings have important implications for marketers and website designers seeking to enhance their green product sales by developing innovative website designs that incorporate technological integration and build trust with consumers. Both markers and website designers can use these insights to optimize their website design strategies, leading to increased conversion rates.

## 1 Introduction

Improving trust is widely considered among marketers and website practitioners as building credibility and transparency with green consumers [1,2]. Green products often face skepticism from consumers who are concerned about greenwashing or marketers making false environmental claims [3,4]. By including trust antecedents on the website, such as eco-certifications, transparent information about environmental impact, and social responsibility initiatives, website designers can help build credibility and establish trust with consumers [5]. Incorporating trust and technology integration has been acknowledged as a critical driver of creating a sense of community [6]. Innovative website design can play a role in building particular trust characteristics and facilitating trust transfer, which can influence how green consumers convert [7]. Technology integration of innovative website design can also improve the quality of information provided to green consumers [8]. By using technologies such as social media integration and design techniques, website designers can create a website that is more effective at communicating information, building trust, and fostering a sense of community with green consumers [9]. Technology integration of innovative website design is an essential part of building trust and getting consumers to respond to the call-to-action [10]. The ability to identify and integrate technology for innovative website design is a key strategic essence for marketers and website builders. Marketers and website designers are faced with many constraints in light of low engagement and low credibility, indicating a need to take advantage of the existing website, trust being one such factor. This research evaluates technology integration of innovative website design to improve site navigation. In turn, it examines the concept of trust and assesses the resulting implications for website designers. In this context, this research poses a key research question: how does technological integration of innovative website design affect green consumer conversion to buying green products when trust matters?

There is an abundance of literature pointing to the importance of particular trust antecedents as determinants of the possible conversion of green consumer response to buying green products, for example, website usability [11] and website quality requirement [12]. However, the trust-building mechanism is constrained by user perception [13]. The design must be visually appealing, easy to understand, and match the overall tone and style of the website. Users may not trust a website if they perceive the trust-building mechanisms as intrusive, overly aggressive, or irrelevant. The role of anticipated website usability and anticipated website quality-assured requirements is pivotal as mediating variables in the relationship between consumer interactions with the site and their trust in the environmental benefits of green products. Anticipated website usability, which emphasizes the clarity and accessibility of information, not only facilitates user navigation but also mediates the consumer's educational journey, directly impacting their trust in the information provided. Concurrently, anticipated website quality-assured requirements, focusing on the technical performance of the website, mediates the perceived reliability and professionalism of the platform, which are critical components influencing user trust. However, it's critical to note that the initial introduction did not address these mediating variables explicitly. Integrating AWU and AWQAR into our analysis is essential as they bridge the gap between user interface/experience and the psychological aspect of consumer trust. By understanding and optimizing these variables, businesses can strategically enhance the effectiveness of their platforms in promoting the environmental advantages of green products, thereby building a stronger, more trusting consumer base. Thus, it is important to focus on how trust can be established in order to encourage defined conversion actions on the website.

Although trust is critical, it is necessary to incorporate trust antecedents into website design to enhance the overall success and impact of green products. Innovative website design must consider how it manifests and functions to connect the trustor and trustee, thereby facilitating green product conversion. This research examines how trust antecedents and trust transfer are integrated with e-commerce websites, with a focus on how technology integration of innovative website design drives this process. Prior research has extensively focused on identifying the role of trust in decision-making and intention formation. Studies have consistently shown that trust has a significant impact on consumer behavior, influencing their perception of product quality [14], brand loyalty [15], and purchase intentions [16]. Teo & Liu (2007) find positive characteristics of trustees (i.e., perceived reputation and system assurance) and trustors (i.e., propensity to trust) as increasing consumer trust to buy products on the e-commerce website [17]. When it comes to the establishment of strong brand trust, Liu et al. (2018) find that trust shared among customers-to-customers (C2C) and customer-to-marketers (C2M) increases the level of engagement [18]. Cheng et al. (2019) found that, when viewed through the lens of trust transfer, trust performance is activated when specific antecedents of trust, such as particularized trust towards e-commerce members, and system trust towards e-commerce apps are well-placed [19]. According to Zhao et al. (2019), social support theory can explain the development of trust in sellers. Specifically, when social support in the form of information increases, so does trust [20]. One research gap is the need for further examination of the specific design features that promote trust in websites. This research differs from the aforementioned studies by considering design elements such as perceived usability as important factors in building trust. There is still a need to investigate which specific design features can lead to users perceiving a green product positively before making a purchase.

Against this backdrop, the aim of this research is to investigate the effect of technological integration on innovative website design in the context of promoting the conversion to buy green products, while considering the role of trust. This research builds on the integrated view of trust transfer in website design by incorporating technology integration into the innovative website design process. The study evaluates the effect of the trust-building mechanism on the potential conversion to buy green products. The empirical analysis is conducted based on data collected from Thai users.

The contribution of research on the integration of technology in innovative website design to promote the conversion of green products lies in its potential to enhance the perceived trustworthiness of websites and positively impact green consumer response. Through the incorporation of features such as customer reviews (supported by an endorsement by other users), innovative website design can build trust with green consumers and increase their likelihood of engaging in positive behaviors such as purchasing green products. Furthermore, although constructs such as usability and information quality may not be statistically significant in the structural analysis, the study of trust and technology integration in innovative website design can still help to identify specific design features that promote trust and positively impact Thai consumer response. This knowledge can inform the development of effective strategies for promoting green products online and contribute to the broader fields of e-commerce, marketing, and environmental sustainability.

The rest of the document is structured in the following manner. Initially, we provide additional information on the relevant literature and theory that underpins the conceptual framework. Subsequently, we delve into specifics about our data collection, statistical approach, and the variables that we measured. Lastly, we analyze our results and address any constraints, as well as potential areas for future research.

## 2 Theory and hypothesis development

The literature review covers three main areas: (1) technology integration of innovative website design, (2) trust antecedents, such as endorsement by other users, perceived website usability, quality assurance, anticipated user experience, and user trust towards innovative website design, and (3) user trust and the conversion of user response to green product purchases.

Within the extant body of literature, the confluence of consumer trust, website conversion rates, and their relation to green product e-commerce platforms remains sparsely trodden, revealing a distinct research gap that this study aims to fill. Current scholarly dialogue primarily orbits around trust and website conversions in broad e-commerce settings, with scant focus on the burgeoning domain of environmentally sustainable products [3,21,22]. Moreover, the influence of innovative website design, especially its technological aspects, on consumer behavior is a nascent field of inquiry in this context (Cyr, Head, & Ivanov, 2006).

Our research introduces novelty by interlinking these spheres, proposing an unprecedented analytical framework that scrutinizes the impact of advanced technology integrations in website design on consumer trust and conversion rates, specifically tailored to green product purchasing. This approach not only extends but also refines existing conceptualizations of e-commerce trust and conversion models [23], by underscoring the role of website design innovation—a critical element surprisingly underrepresented in current discourse.

Furthermore, while trust development in online environments is well-theorized [19,24], our study pioneers the exploration of how this translates into the green product market, an area gaining immense momentum yet not fully understood in digital commerce literature. By elucidating the technological facets of website design that bolster consumer trust and, consequentially, website conversions in this sector, we provide fresh, practical insights that are immediately applicable for businesses advocating sustainability.

### 2.1 Technology integration of innovative website design

In this research context, technological integration of innovative website design refers to the process of incorporating advanced digital technologies and techniques in the creation of web-based applications to deliver a user experience [[25], [26], [27]]. This involves using techniques such as responsive web design [28], interactivity [29], social media integration [30], user-generated content [31], and continuous updates to provide high-quality information, ease of use [32], and visually appealing design [33]. The aim of technological integration of innovative website design is to increase customer satisfaction, establish trust, and enhance the overall effectiveness of a website [34,35]. The recent cross-sectional study by Ref. [36] shows that creative expression on the website may be analogous to consumer engagement in the digital retail space. The aesthetic components of web design, including color schemes and imagery, play a crucial role in shaping a consumer's trust and purchase intent towards a product [37]. The appeal of a website's color scheme significantly contributes to fostering trust in a website, and it is noteworthy that consumer perceptions regarding color appeal may vary across different cultural backgrounds [38,39]. Incorporating technology integration into innovative website design requires considering key factors. Trust antecedents, such as endorsement by other users, perceived website usability, quality assurance, anticipated user experience, and user trust towards innovative website design, are reviewed and discussed.

### 2.2 Anticipated website usability

Anticipated website usability refers to the user's expectation or perception of the ease of use and effectiveness of a website before they actually use it [40]. It is the user's prediction or anticipation of how easy it will be to navigate the website, find the desired information or products, and accomplish their goals on the website [41,42]. Technology integration of innovative website design can have a positive impact on anticipated website usability [35]. Innovative website design often involves the incorporation of advanced technological features and functionalities, such as intuitive navigation, personalized recommendations, and responsive design [43]. These features can enhance the overall user experience, making the website easier and more enjoyable to use. As a result, users may anticipate that the website will be highly useable and user-friendly, which can lead to increased trust on the website and its content [44]. Additionally, innovative website design can also improve website speed and performance, which can further enhance anticipated website usability [45,46]. It comes to the following-developed hypothesis:

H1 Technology integration of innovative website design positively affects anticipated website usability.

Anticipated website usability is linked to user trust towards innovative website design because when users perceive a website as easy to use, they are more likely to trust it [41]. If a website is difficult to navigate, users may become frustrated and lose trust in the website's ability to provide them with the information or services they need. On the other hand, a well-designed website with intuitive navigation and relevant content can enhance the user experience and foster trust in the website's reliability and competence [47]. Moreover, if users can find what they are looking for quickly and easily, they are more likely to perceive the website as credible and trustworthy [48]. Therefore, anticipated website usability can positively affect user trust towards innovative website design by enhancing user experience and reducing perceived risk [49]. Hence, the following hypothesis was formulated:

H2 Anticipated website usability positively affects user trust towards innovative website design.

### 2.3 Anticipated website quality-assured requirements

Anticipated website quality-assured requirements refer to the expected standards and processes that must be implemented to ensure that a website meets certain quality criteria [12,19]. Innovative website designs may also introduce new functionalities, such as user-generated content, social media integration, or real-time communication, that require additional quality-assured requirements to ensure their proper functioning [50]. For instance, user-generated content may require additional measures to protect against inappropriate content, while real-time communication may require additional security measures to protect user data [51]. The integration of technology in innovative website design can affect anticipated website quality-assured requirements by requiring the implementation of new standards and processes [44,52]. The following assumption was made:

H3 Technology integration of innovative website design positively affects anticipated website quality-assured requirements.

In the current study, the website's quality-assured requirements are aimed at creating the best website visiting experience while ensuring trust, particularly with regards to security and privacy. The challenge of trust amplifies on online platforms due to associated security risks, making it difficult for users to trust e-commerce and social commerce portals [53,54]. The expectations of a website's quality-assured features can greatly influence the level of trust a user has on the website. Anticipated website quality-assured requirements can be linked to the concept of quality-assured shared information discussed by Cheng et al. (2019), which emphasizes the accuracy, correctness, timeliness, and usefulness of the information shared by members on e-commerce website [19,55]. Previous research by Hooda et al. (2022); S. Kim and Park (2013); Oliveira et al. (2017); Sarkar et al., 2020) demonstrated the substantial impact of information quality on the formation of trust, suggesting that websites that prioritize and ensure quality-assured requirements are likely to foster greater trust among users [[55], [56], [57], [58], [59]]. Therefore, it is reasonable to expect that anticipated website quality-assured requirements could positively influence user trust in a similar manner as information quality does in the context of e-commerce website design. When a website meets or exceeds a customer's expectations for quality, it can help to build trust and increase the likelihood of conversion.

H4 Anticipated website quality-assured requirements positively affect user trust towards innovative website design.

### 2.4 User trust towards innovative website design

#### 2.4.1 User trust and the conversion of a green product purchase

**Fig. 1:**
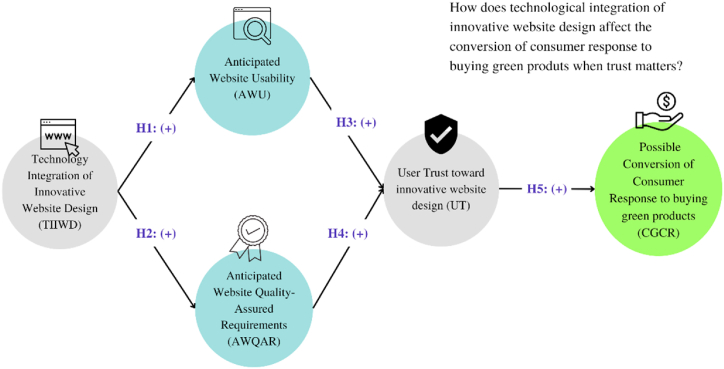
Conceptual framework.

User trust is an important factor when it comes to the conversion of a green product purchase. When customers are considering purchasing a green product, they want to be sure that the product is actually eco-friendly and that the claims made by the company's website are true. Fig. 1 depicts that the conceptual model highlighting the importance of trust-building factors and the technology integration of innovative website design in building user trust and increasing the conversion of green product purchase.

The current research defines the conversion of a green product purchase on the innovative website design as the process of a website user transitioning from a potential customer to an actual customer by making a purchase of an environmentally friendly or sustainable product on the website. This process is influenced by various factors, including the user's trust on the website and the product being offered. If the user trusts the website and perceives the product to be environmentally friendly, they are more likely to make a purchase, thereby converting from a potential customer to an actual customer.

Previous studies suggest that when users have a higher level of trust towards an innovative website design, they are more likely to engage in positive behaviors towards the website, such as making a purchase [19,[60], [61], [62], [63]]. Users become increasingly aware and concerned about environmental issues, including carbon emissions. They tend to trust firms that are transparent and proactive in reducing their carbon footprint [64]. In the context of green products, if users trust the website and its information, they are more likely to purchase green products [65,66]. This is because trust reduces the level of risk perceived by the user, making them feel more comfortable in making a purchase decision [67]. Additionally, users who trust a website are more likely to return to the website in the future and recommend it to others, which can lead to increased sales and positive brand reputation for the organization [12]. Therefore, the positive relationship between user trust as trust transfers towards innovative website design and the conversion of green product purchase can be critical. Hence,

H5 User trust towards innovative website design increases the conversion of a green product purchase

## 3 Method

This section covers two key aspects of research methodology: data collection and sampling and variable operationalization.

### 3.1 Data collection and sampling

This study employed a quantitative research design. Data was collected through an online survey since the research of interest is regarding innovative website design (i.e., e-commerce website). Google Form and Quick Response (QR) were used to conduct an electronic survey for this research. The survey collected responses from representatives in all geographical areas of Thailand, namely: North, Northeast, Central, Eastern, Western, and Southern regions. To determine the sample size for the survey, the research identified the whole population of the study as a known population. This is because there is a finite limit to the number of people who may potentially purchase an order on an e-commerce website. This research recognized the importance of grounding this discussion in real-world data to provide context and enhance the credibility of the research analysis. In Thailand, there are 36.60 million e-commerce shoppers who spent about $18.97 billion in 2021. This means each person spent roughly $518 on online consumer goods. Additionally, about 65.1 % of these shoppers made purchases via their mobile phones [68]. However, this research employed this figure of 36.6 million e-commerce consumers as the whole population for sample size calculation only, providing a pragmatic basis for statistical analysis while acknowledging the ever-expanding and fluid nature of the online shopping market.

The formula with the finite population correction is:

$$n=\frac{\frac{z^{2}\times p\left(1-p\right)}{e^{2}}}{1+\frac{z^{2}\times p\left(1-p\right)}{e^{2}N}}$$

where ‘*z*' represents the z-value associated with the desired confidence level (1.96 for a 95 % confidence level), ‘*p*' is the estimated proportion of the population with the characteristic of interest (often conservatively set at 0.5 or 50 % to maximize the sample size), ‘*e*' is the level of precision or margin of error (such as 0.05 for a 5 % margin), and ‘*N*' is the total population size [69]. For a population of 36.60 million, the calculation suggests a minimum sample size of approximately 384. This size would be adequate to estimate the proportion of users with certain characteristics or behaviors within the target population.

To recruit 384 potential users, this research utilized online panels, such as Facebook groups related to Shopee, Lazada, and other e-commerce websites in Thailand. In doing so, the research team contacted the group administrators to obtain permission before posting the online survey on the groups. Purposive sampling is a non-probability sampling technique that involves selecting individuals or cases that meet specific criteria. This method can be particularly useful when studying innovative website design and website conversion because it allows researchers to select participants who have specific characteristics or experiences that are relevant to the research question. The potential respondents were selected with some inclusion criteria:

• They must be over 18 years old. This is followed by the ethical consideration.
• They must have experience using e-commerce websites. This criterion is set up on the first page of the online survey, with all explanations clarified. If they do not have experience using e-commerce websites, they are asked to return the survey.

### 3.2 Variable operationalization

All measures in this study have been modified and validated in previous studies. Appendix A shows the list of questions brought to evaluate users' website conversion. Several modifications have been implemented to align with the current research context. After translation, the questionnaire was back-translated into the Thai language by a different set of bilingual experts. Discrepancies between the original and back-translated versions were resolved by re-evaluating and adjusting the Thai translation as necessary. In the initial phase of our research, we conducted a pilot test involving 30 participants from the target population to ensure the clarity, understandability, and relevance of the questionnaire items for native Thai speakers. This crucial step allowed us to gather preliminary feedback on the questionnaire's content and structure, enabling us to identify any ambiguous or challenging items. To maintain the integrity and robustness of our research design, we made a deliberate decision not to include the 30 respondents from the pilot phase in the main study. This approach was adopted to ensure that the data collected in the main study was entirely fresh and unbiased, preventing any possible contamination due to prior exposure to the questionnaire. The items in all constructs were rated on a five-point Likert scale. There are three sets of scales applied: (1) agreement, (2) anticipation, and (3) tendency. The first set of the Likert scale was the agreement level, where ‘1′ indicated ‘strongly disagree’ and ‘5′ indicated ‘strongly agree.’ The second set of the Likert scale was the anticipation level, where ‘1′ indicated ‘strongly unanticipated’ and ‘5′ indicated ‘strongly anticipated.’ The third set of the Likert scale was the tendency level, where ‘1′ indicated ‘unlikely’ and ‘5′ indicated ‘likely.'

Technology integration of innovative website design (TIIWD) is the extent to which the incorporation of advanced technological features into website design with the aim of enhancing website conversion. The four-item scale was developed from a variety of prior studies such as [[70], [71], [72]]. These aspects included (TIIWD1) virtual reality, (TIIWD2) augmented reality, (TIIWD3) chatbots, and (TIIWD4) social media integration.

Anticipated website usability (AWU) is the extent to which users perceive that a website will be easy to use and navigate before actually using it. It involves users' expectations about the website's interface, features, and overall design, as well as their anticipated ability to accomplish their goals and tasks on the website. The four-item scale was adopted with minor change from Refs. [11,73]. These aspects were (AWU1) learnability for the first-time user, (AWU2) understandability (e.g., language support), (AWU3) navigability (e.g., useful menu bar, search engine, link to home page etc.), and (AWU4) ease of use (e.g., clear fonts, relevant graphics etc.).

Anticipated website quality requirement is the expected level of quality that users anticipate when visiting a website. The four-item scale was developed from Refs. [12,44,52]. These aspects included (AWQR1) users’ privacy and terms and conditions, (AWQR2) security of personal information, (AWQR3) data protection and transparency, and (AWQR4) autonomy to control personal information.

User trust in innovative website design is the level of confidence and reliance that a user has in the trustworthiness and credibility of an innovative website. The four-item scale was integrated from Refs. [13,74,75]. To measure user trust in innovative website design, these aspects included (UT1) website expertise for customer needs, (UT2) website safety and trustworthiness, (UT3) website content reliability and consistency, and (UT4) website loyalty to users.

Conversion of consumer (or user) response to green product purchase is the process of changing consumers’ attitudes and behaviors towards environmentally friendly products, leading them to purchase such products. The four-item scale used to measure website conversion was modified and self-developed based on previous research by Ref. [67]. These aspects of website conversion included (CCR1) consideration of website visiting, (CCR2) user satisfaction, (CCR3) positive feeling about the website design, and (CCR4) conversion to purchase.

## 4 Data analysis and results

Covariance-based structural equation modelling (SEM) is a statistical technique that allows the underlying model and complex relationships between variables to be specified and tested [[76], [77], [78]]. The analysis was carried out using SPSS AMOS 28 and SPSS Statistics 28. It is considered appropriate for studying website design and conversion of user response because it enables this paper to model the interrelationships among multiple variables and understand how they collectively influence the outcome of interest. Website design and the conversion of consumer response are complex phenomena that involve multiple variables, such as website usability, visual design, and trust. These variables are interrelated and can impact each other, making it difficult to assess their individual effects on the conversion of consumer response. SEM is particularly useful in studying website design and conversion of consumer response because it allows researchers to specify a theoretical model that captures the relationships between the various variables involved. By specifying these relationships, SEM can help identify the key drivers of conversion and how they interact with each other. This paper conducted several procedural steps as follows.

**First**, descriptive analysis was conducted. Table 1 describes sample characteristics. The data presents information on the demographic profile, education, income, region, and website preference of a group of individuals. In terms of gender, 57.3 % are female, while 42.7 % are male. In terms of age, the largest group is aged 18–24 (44.8 %), followed by those aged 55–64 (23.2 %). There is only one person (0.3 %) over the age of 65. Most respondents have a Bachelor's degree (71.4 %), followed by those with a Master's degree (11.7 %). In terms of income, the largest group earns between 10,001–15,000 Thai Baht (23.2 %), followed closely by those earning 15,001–20,000 Thai Baht (21.6 %). The majority of respondents visit e-commerce websites (52.3 %), followed by blogs (21.9 %) and government websites (8.1 %). Geographically, the largest group of respondents is from the Northeastern region (58.1 %), followed by the South (20.8 %). The smallest group is from the West (2.9 %).

**Table 1:** Sample characteristics.

Demographic	Category	Total	Percentage (%)
Gender	Male	164	42.70 %
	Female	220	57.30 %
Age	18–24	172	44.80 %
	25–34	33	8.6 %
	35–44	53	13.30 %
	45–54	36	9.40 %
	55–64	89	23.20 %
	Over 65	1	0.30 %
Education	High school graduate, diploma, or the equivalent	53	13.80 %
	Bachelor's degree	274	71.40 %
	Master's degree	45	11.70 %
	Doctorate	1	0.30 %
	Others	11	2.90 %
Monthly Income (THB)	Less than 5000	13	3.40 %
	5001–10,000	53	13.80 %
	10,001–15,000	89	23.20 %
	15,001–20,000	83	21.60 %
	20,001–25,000	38	9.90 %
	25,001–30,000	26	6.80 %
	30,001–35,000	19	4.90 %
	35,001–40,000	12	3.10 %
	40,001–4,5000	20	5.20 %
	45,001–50,000	6	1.60 %
	50,001–55,000	0	0
	More than 55,001	25	6.50 %
Region	North	34	8.90 %
	Northeastern	223	58.10 %
	Central	24	6.30 %
	South	80	20.80 %
	West	11	2.90 %
	Eastern	12	3.10 %
What website do you usually visit?	Government	31	8.10 %
	E-commerce	201	52.30 %
	Magazine	30	7.80 %
	Blogs	84	21.90 %
	Others	38	9.90 %

**Second**, to assess the presence of a single dominant factor in a dataset, the Harman single-factor test was conducted. Its result showed that the total variance explained by a single factor in the Harman single-factor test was 41.661 %, which is below the recommended threshold of 50 % [79,80]. Thus, it can be claimed that this data does not exhibit any issues with common method bias.

**Third**, to evaluate the quality of reflective measurement models estimated by covariance-based SEM, both in in terms of reliability and validity [81], confirmatory factor analysis (CFA) was carried out. Confirmatory Factor Analysis (CFA) is a statistical technique that is used to test the hypothesis that a set of observed variables are measuring an underlying construct or latent variable [78,82,83]. During the CFA process, Table 2 exhibits the construct validity and reliability of the measurement model. The results of construct validity showed that factor loadings yielded between 0.651 and 0.891, which are above the recommended threshold of 0.6 or 60 %. Thus, the overall pattern of factor loadings across all variables can be used to understand the underlying factors that contribute to the observed data. Composite reliability values between 0.822 and 0.904 are considered good as they are higher than the recommended cutoff of 0.7 or 70 % for measuring the association between indicators that measure the same construct [77,83]. Thus, it can be claimed that the indicators are internally consistent and reliable. To assess whether the indicators of a construct are converging to explain their variance [82], the Average Variance Extracted (AVE) is used. In this study, the AVE values ranged from 0.536 to 0.704, which is above the recommended threshold of 0.5 or 50 % of variance. The Cronbach's Alpha (α) values ranged from 0.822 to 0.903, indicating a high level of internal consistency within the employed scale. Such values, falling between good to excellent on the commonly accepted interpretation scale, demonstrate the potential reliability of the measurement instrument in assessing the intended construct consistently, thereby providing a more robust understanding of the research findings' validity and reliability. The values of the variance inflation factor are well below the commonly used thresholds of 5 or 10, which suggests that, for most of your variables, multicollinearity is not severely inflating the variance of the coefficients in the model.

**Table 2:** Construct validity and reliability.

Construct	Parameter	Bias-corrected bootstrap methods				AVE	CR	Cronbach's alpha	Variance Inflation Factor
		Standardized estimate	Lower	Upper	*P*-value				
Anticipated Website Quality Requirement (AWQR)	AWQR4	0.859	0.794	0.908	0.004**				2.283
	AWQR3	0.727	0.649	0.802	0.003**				2.179
	AWQR2	0.837	0.781	0.891	0.003**				2.179
	AWQR1	0.767	0.693	0.829	0.004**	0.639	0.876	0.861	2.004
User Trust in Innovative Website Design (UT)	UT1	0.763	0.687	0.819	0.006**				2.398
	UT2	0.83	0.761	0.876	0.006**				3.226
	UT3	0.891	0.852	0.921	0.006**				4.878
	UT4	0.866	0.819	0.904	0.004**	0.704	0.904	0.903	4.016
Conversion of Consumer Response to Green Product Purchase (CCR)	CCR1	0.735	0.65	0.805	0.003**				2.165
	CCR2	0.866	0.808	0.918	0.003**				3.984
	CCR3	0.729	0.656	0.806	0.003**				2.151
	CCR4	0.705	0.622	0.784	0.003**	0.580	0.846	0.851	1.992
Technology Integration of Innovative Website Design (TIIWD)	TIIWD4	0.765	0.677	0.828	0.003**				2.304
	TIIWD3	0.79	0.723	0.845	0.003**				2.381
	TIIWD2	0.742	0.659	0.812	0.003**				2.024
	TIIWD1	0.651	0.562	0.732	0.004**	0.546	0.827	0.839	1.626
Anticipated Website Usability (AWU)	AWU1	0.698	0.616	0.773	0.002**				2.079
	AWU2	0.732	0.658	0.801	0.002**				3.663
	AWU3	0.733	0.665	0.791	0.003**				2.257
	AWU4	0.763	0.674	0.82	0.007**	0.536	0.822	0.822	3.012

Note: p-value <0.01**.

**Fourth**, Table 3 illustrates how the assessment of discriminant validity determines the extent to which a construct is distinct from other constructs in the structural model, based on empirical evidence. The HTMT values in the study ranged from 0.335 to 0.801, which met the threshold value of below 0.90 proposed by Henseler et al. (2015). Therefore, it can be concluded that there was no issue of multicollinearity, and the presence of discriminant validity can be claimed.

**Table 3:** Heterotrait-monotrait ratio of correlations (HTMT).

	AWU	TIIWD	CCR	UT
**TIIWD**	0.732			
**CCR**	0.607	0.653		
**UT**	0.355	0.418	0.482	
**AWQR**	0.801	0.683	0.655	0.411

**Fifth**, a well-fitting model is a statistical model that accurately represents the degree to which the hypothesized measurement model aligns with the observed data. To evaluate the goodness-of-fit measures, fit indices are commonly used, including Chi-square, CMIN/DF, SRMR, NFI, TLI, CFI, and RMSEA.

The chi-square value of 343.85 with 157 degrees of freedom was found to be significant at a p-value of less than 0.05. Various measures can be used to assess the acceptability of the fit between a hypothetical model and sample data. The minimum discrepancy per degree of freedom (CMIN/DF) ideally should be less than 3 for an acceptable fit according to some studies [25] and less than 5 according to others [69]. In this study, CMIN/DF was 2.19, indicating an acceptable fit.

Additionally, the standardized root mean square residual (SRMR) resulted in a value of 0.0429, which was below the cutoff value of 0.08. A root mean squared error (RMSEA) point value of less than 0.08 is considered a good fit as suggested by Ref. [83]. The RMSEA value was 0.056, indicating a better fit between the model and the observed data.

Furthermore, a structural equation model is considered acceptable if the Comparative Fit Index (CFI) value is greater than 0.9 and the Tucker Lewis index (TLI) value is above 0.9 according to Ref. [84]. In this study, both CFI and TLI values were 0.958 and 0.949, respectively, indicating a better fit between the model and the observed data. The overall results of the CFA were satisfactory, indicating an acceptable fit between the hypothesized model and the observed data. The evaluation of the structural model could be performed in the next step.

**Sixth**, the goodness of fit (GOF) of the structural model was evaluated. The model produced a significant Chi-square value (CMIN = 571.254; DF = 163; and p-value <0.001), with a CMIN/DF ratio of 3.505. Other fit indices, such as RMSEA = 0.08 and CFI = 0.908, were also considered and found to indicate an acceptable fit [85]. Therefore, it can be concluded that the proposed model effectively explains the technology integration of innovative website design. Moving forward, the next section will discuss hypothesis testing in a timely manner.

## 5 Discussions

This section addresses three main areas of discussion: hypothesis testing, the argument between the current research findings and previous studies, and the implications of the results.

### 5.1 Hypothesis testing

The bias-corrected percentile method was chosen for analysis in SEM because it provides more accurate estimates of the confidence intervals for the model parameters compared to other methods, such as the standard error-based method or the percentile bootstrap method. Table 4 displays the results of the structural model using the bias-corrected percentile method for estimation.

**Table 4:** Structural model result.

H	Causal Relationships			Bias-corrected bootstrap methods				Squared Multiple Correlations	Results
				Standardized Estimate	Lower	Upper	*P*-value		
H1	Technology Integration of Innovative Website Design	→	Anticipated Website Usability	0.828	0.73	0.914	0.003**	0.686	Supported
H2	Technology Integration of Innovative Website Design	→	Anticipated Website Quality Requirements	0.78	0.677	0.858	0.003**	0.609	Supported
H3	Anticipated Website Usability	→	User Trust in Innovative Website Design	−0.089	−0.43	0.194	0.562	0.208	Unsupported
H4	Anticipated Website Quality Requirements	→	User Trust in Innovative Website Design	0.154	−0.025	0.357	0.081^+^		Unsupported
H5	User Trust in Innovative Website Design	→	Conversion of Consumer Response to Green Product Purchase	0.203	0.075	0.318	0.014*	0.571	Supported

**Note:** p-value <0.1^+^; p-value <0.05*; p-value <0.01**.

The results of the structural model indicate that the integration of technology into innovative website design has a significant positive effect on expected website usability at a level of 0.3, supporting Hypothesis 1 (β=0.828;p−value=0.003). Additionally, the integration of new technology into innovative website design has a positive effect on expected quality-assured website requirements, supporting Hypothesis 2 (β=0.78;p−value=0.003). Furthermore, users were not found to be more likely to trust innovative website design when they perceived the site as easy to use, which does not support Hypothesis 3 (β=−0.089;p−value=0.562). However, anticipated website quality-assured requirements, as a trust antecedent, increased user trust towards innovative website design, and thus, Hypothesis 4 was supported (β=0.154;p−value=0.003). Finally, the results revealed that a user's trust in a website's innovative design made them more likely to purchase a green product, which supports Hypothesis 5 (β=0.203;p−value=0.014). SPSS AMOS provides squared multiple correlations for each endogenous variable in the model, which can be interpreted as the proportion of variance in the dependent variables that is explained by the independent variables. This is a measure of effect size. This research employed squared multiple correlations to explain the model. It is estimated that 68.6 % of the anticipated website usability and 60.9 % of the anticipated website quality requirements can be explained by the technology integration of innovative website design. In a structural analysis, a set of anticipated website usability and anticipated website quality requirements can predict 20.8 % of the variance in user trust in innovative website design. Additionally, it is estimated that the predictors of consumer response to green product purchase can explain 57.1 % of its variance.

Mediation analysis is a statistical method that facilitates the evaluation of the association between two variables by scrutinizing the contribution of a mediator variable in explaining the relationship. The mediator variable is assumed to exist between the independent and dependent variables. The analysis evaluates the direct, indirect, and overall effects of the mediator variable on the relationship between the two variables. Table 5 shows the results of mediation analysis. Variance accounted for (VAF) is a common method for interpreting mediation analysis results. The analysis yielded a VAF value of around 23.3 %, indicating a VAF between 20 % and 80 % [82]. This is called partial mediation. This result indicated that 23.3 % of the total effect of technology integration of innovative website design on user trust in innovative website design is *partially* accounted for by anticipated website quality requirements. No other mediation relationships were found, and therefore, no additional mediation results were claimed.

**Table 5:** Mediation analysis.

Mediation relationships	A	B	C	Indirect Effect (AxB)	Total Effect (AxB + C)	Variance Accounted For (VAF)	Mediation Results
TIIWD --- > AWU --- > UT	0.828**	−0.089 (n.s.)	0.396**	N.A.	N.A.	N.A.	No mediation: this is just a common cause
TIIWD --- > AWQAR --- > UT	0.78**	0.154^+^	0.396**	0.120	0.516	0.233	Partial mediation: but weak evidence
AWU --- > UT --- > CCR	−0.089 (n.s.)	0.203*	0.023 (n.s.)	N.A.	N.A.	N.A.	No mediation: this is just a single effect
AWQAR --- > UT --- > CCR	0.154^+^	0.203*	0.236**	0.031	0.267	0.117	No mediation: rather claimed that it is a common effect on CCR

**Note:** p-value <0.1^+^; p-value <0.05*; p-value <0.01**; p-value <0.001***; n.s. = p-value is not significant; N.A. = not applicable.

After confirming the proposed hypotheses, the current research findings were compared to previous studies to examine their consistency and differences in the next section.

### 5.2 The discussion of the argument between the current research findings and previous studies

The small but growing body of literature on innovative website design for website conversion emphasizes its role as a pivotal factor in user trust. The first objective was to investigate the effect of technology integration on innovative website design in the context of promoting the conversion to buying green products while considering the role of trust. The results supported the idea that when users or consumers perceive the integration of innovative website design technology, they are more likely to trust the design and convert to purchasing products on the website.

The key findings can be argued as follows. The findings of this paper suggest that incorporating innovative website design technology can lead to better user experiences, specifically in terms of anticipated website usability. The positive relationship found between technology integration and anticipated website usability aligns with previous research in the field, such as [35,41,42]. Innovative website design can have a significant impact on website usability by improving accessibility [41], user interface, functionality, and engagement. By investing in innovative website design technologies, businesses can create a more user-friendly and engaging website, which can ultimately lead to better business outcomes. As such, website design and website usability should be considered together when developing a website, as they are closely related and can have a significant impact on each other.

The research finding suggested that innovative website design is positively related to anticipated website quality-assured requirements. While there may not be any specific studies in the literature, such as [12,19,50] that provide direct evidence to support this argument, it is still a valid argument that can be made based on general principles of website design and user experience.

When considering website usability and quality requirements to increase user trust, anticipated website usability was not found to have a statistically significant effect, while anticipated website quality requirements were found to be what Thai users look for. This may be because of poorly designed interfaces, user demographics, and competing trust factors (e.g., brand reputation, user reviews, etc.), tech-savvy users might find usability less of a concern compared to older individuals who are not as familiar with digital interfaces, which could lead to a non-significant finding. This finding, albeit localized, prompts a broader exploration into how website interfaces, in general, impact user trust globally. The digital era has ushered in an age where website interfaces serve as the primary point of contact between businesses and consumers. The concepts of anticipated website quality and anticipated website quality requirements have emerged as critical determinants in building user trust. However, the extent to which each of these variables influences user trust and facilitates consumer engagement remains a point of contention [41]. propose an integrative approach suggesting that both anticipated website quality and anticipated website quality requirements are not mutually exclusive but complementary in building user trust. A study by Ref. [19] demonstrated that websites scoring high on both usability and quality assurance metrics enjoyed higher levels of sustained user trust. This view suggests a synergistic effect where usability draws users in, while quality assurance retains their trust over time.

Innovative website design has become increasingly important for companies as they strive to attract and retain customers in an ever-competitive online marketplace. However, the effectiveness of innovative website design in building user trust and promoting the conversion of green product purchases remains an open question. One way that innovative website design can help build trust with users is by providing a seamless and intuitive user experience. When users can easily navigate and find the information they need on a website, they are more likely to trust the company and its products. Additionally, innovative website design elements, such as interactive features and engaging visuals, can help capture users' attention and build a sense of connection with the company and its brand. However, it is unclear whether innovative website design has a direct effect on the conversion of green product purchases. This research results suggested that user trust in innovative website design increases the conversion of green product purchase. This aligns with the previous study by Shaouf et al. (2016) supporting that website design, including factors such as aesthetics, functionality, and information quality, had a positive effect on green purchase intention. Specifically, the study found that perceived aesthetics and perceived functionality of the website were positively related to perceived value of green products, which in turn had a positive effect on green purchase intention. Another study by Lu et al. (2017) found that website design elements, such as visual appeal, navigation, and content quality, had a positive effect on consumer trust. Additionally, the study found that consumer trust had a positive effect on the purchase intention of green products. Therefore, marketers and website designers looking to promote the conversion of green product purchases should take a holistic approach, considering all of these factors (e.g., designing an easy-to-navigate website and utilizing Search Engine Optimization (SEO) strategies) and working to build trust and credibility with users across all touchpoints.

### 5.3 Implications

The integration of innovative website design in promoting green products can have a significant impact on green consumer conversion to buying green products when trust matters. Innovative website design elements, such as engaging visuals and interactive features, can capture users’ attention and build a sense of connection with the company and its brand [35,70,86]. This can help to build trust with users, which is a critical factor in promoting the conversion of green product purchase.

The present study results suggested that user trust in innovative website design increases the conversion of green product purchase, which highlights the importance of innovative website design in promoting green products. Marketers and website designers can use innovative website design to enhance the user experience and provide a seamless and intuitive experience for green consumers, thereby building trust and promoting green product purchases.

Moreover, website designers should ensure that their website design is mobile-friendly, as more and more consumers are using mobile devices to browse the internet and make purchases. A mobile-friendly website can improve the user experience and increase the likelihood of green consumers making a purchase. The integration of innovative website design in promoting green products can have a significant impact on green consumer conversion to buying green products when trust matters. Marketers that prioritize innovative website design and focus on building trust with green consumers are likely to see an increase in green product purchases and contribute to a more sustainable future.

### 5.4 Conclusion

This section covers three key aspects of the study's findings: the primary conclusion, its limitations, and areas for future research.

This paper investigates how the integration of technology influences the impact of innovative website design in promoting the conversion to buying green products, with attention to the role of trust. The concept of user trust in innovative website design is introduced in this paper, which can better reflect the characteristics of e-commerce websites. The research also identifies two trust antecedents of a website starting from its design: website quality-assured requirements and usability. These are trust-building website characteristics in the e-commerce community. Website designers and marketers could refer to these antecedents of trust to better build their e-commerce website for a better user journey. Additionally, we confirm the partial mediating role of anticipated website quality requirements in the relationship between technology integration of innovative website design and user trust in innovative website design. Moreover, we provide an integrated model of user-trust establishment in innovative website design to increase website conversion, offering alternative explanations about how user trust influences their behaviors on e-commerce websites.

Although this study has provided valuable insights into user trust in innovative website design, it is essential to note its limitations. *First*, the data was collected only in Thailand, limiting the generalizability of the findings to other cultural contexts. Future research should consider expanding the sample to include respondents from other regions. *Second*, while this study identified two antecedents of user trust in innovative website design, there may be other antecedents that were not explored in this research. In particular, we recognize the importance of the relationship between anticipated website usability and anticipated website quality requirements, suggesting that this could be an intriguing avenue for future research, potentially offering further valuable insights. Additionally, future research could focus on identifying other factors, including endorsement by other users and user experience, that may influence particularized trust in innovative website design. This is because user experience can help identify user needs, preferences, and pain points, which can inform website design decisions that improve conversion. *Third*, this study measured website conversion of green product purchases as an indicator of possible user behavioral action. Future studies should consider using the percentage of website visitors who take a desired action as a measure of trust performance. This could be done by website experimental design research. *Finally*, this study was conducted at a single point in time (cross-sectional data), limiting its ability to capture changes in user trust and trust-related behaviors over time. Therefore, future research should consider a longitudinal design to better understand how trust evolves in innovative website design over time.

## Data availability statement

Data will be made available on request.

## Ethical approval

Khon Kaen University Ethics Committee for Human Research, Khon Kaen University, Khon Kaen, Thailand, has made an agreement that this study has met the criteria of the Exemption Determination Regulations on November 11, 2021 (HE643227).

## CRediT authorship contribution statement

**Wutthiya Aekthanate Srisathan:** Writing – review & editing, Visualization, Formal analysis, Data curation. **Chavis Ketkaew:** Supervision, Resources, Project administration, Funding acquisition, Conceptualization. **Narissara Jantuma:** Writing – original draft, Data curation. **Phaninee Naruetharadhol:** Writing – review & editing, Writing – original draft, Conceptualization.

## Declaration of competing interest

The authors declare the following financial interests/personal relationships which may be considered as potential competing interests:Phaninee Naruetharadhol reports financial support was provided by the Office of National Higher Education, Science, Research, and Innovation Policy Council. If there are other authors, they declare that they have no known competing financial interests or personal relationships that could have appeared to influence the work reported in this paper.

## Figures and Tables

**Table:** 

Constructs	Items	Sources
Technology Integration of Innovative Website Design (TIIWD)	Please rate the following website features based on your opinion.	[[70], [71], [72]]
	TIIWD1: Virtual reality	
	TIIWD2: Augmented reality	
	TIIWD3: Chatbots	
	TIIWD4: Social media integration	
Anticipated Website Usability (AWU)	To ensure the best website visiting experience and build trust, how highly do you rate and expect the website's usability to be?	[11,73]
	AWU1: Learnability for the first-time user (e.g., help documents, guidelines etc.)	
	AWU2: Understandability (e.g., language support)	
	AWU3: Navigability (e.g., useful menu bar, search engine, link to home page etc.)	
	AWU4: Ease of use (e.g., clear fonts, relevant graphics etc.)	
Anticipated Website Quality Requirement (AWQR)	To help create the best website visiting experience and trust,	[12,44,52]
	AWQR1: It is expected that users' privacy and terms and conditions (T&C) are required.	
	AWQR2: It is expected that security of personal information is required.	
	AWQR3: It is expected that data protection and transparency is required.	
	AWQR4: It is expected that autonomy to control personal information is required.	
User Trust in Innovative Website Design (UT)	UT1: I believe that the website has the skills and expertise to meet most customer needs.	[13,74,75]
	UT2: I believe that it’s safe and trustworthy to access the website.	
	UT3: I believe that the website content is reliable and consistent.	
	UT4: I believe that the website is loyal to all users.	
Conversion of Consumer Response to Green Product Purchase (CCR)	CCR1: I will probably consider visiting the website.	[67]
	CCR2: I will probably be satisfied with the website.	
	CCR3: I feel positive with the website.	
	CCR4: I will probably convert to buying products on the website.	
